# NFKBIE mutations are selected by the tumor microenvironment and contribute to immune escape in chronic lymphocytic leukemia

**DOI:** 10.1038/s41375-024-02224-8

**Published:** 2024-03-15

**Authors:** Alice Bonato, Supriya Chakraborty, Riccardo Bomben, Giulia Canarutto, Giulia Felician, Claudio Martines, Antonella Zucchetto, Federico Pozzo, Marija Vujovikj, Jerry Polesel, Annalisa Chiarenza, Maria Ilaria Del Principe, Giovanni Del Poeta, Giovanni D’Arena, Roberto Marasca, Agostino Tafuri, Luca Laurenti, Silvano Piazza, Aleksandar J. Dimovski, Valter Gattei, Dimitar G. Efremov

**Affiliations:** 1 Molecular Hematology Unit, International Center for Genetic Engineering and Biotechnology, Trieste, Italy; 2 Clinical and Experimental Onco-Hematology Unit, IRCCS Centro Di Riferimento Oncologico, Aviano, Italy; 3 Computational Biology Unit, International Center for Genetic Engineering and Biotechnology, Trieste, Italy; 4 Research Center for Genetic Engineering and Biotechnology, Macedonian Academy of Sciences and Arts, Skopje, North Macedonia; 5 Division of Hematology, Ferrarotto Hospital, Catania, Italy; 6 Hematology, Department of Biomedicine and Prevention, University of Rome Tor Vergata, Rome, Italy; 7 Hematology and Stem Cell Transplantation Unit, IRCCS Centro di Riferimento Oncologico della Basilicata, Rionero in Vulture, Italy; 8 Division of Hematology, University of Modena and Reggio Emilia, Modena, Italy; 9 Division of Hematology, University Hospital Sant’Andrea, “Sapienza” University of Rome, Rome, Italy; 10 Hematology Unit, Fondazione Policlinico Universitario A Gemelli IRCCS, Rome, Italy; 11 Macedonian Academy of Sciences and Arts, Skopje, North Macedonia

**Keywords:** Chronic lymphocytic leukaemia, Cancer models

## Abstract

Loss-of-function mutations in NFKBIE, which encodes for the NF-κB inhibitor IκBε, are frequent in chronic lymphocytic leukemia (CLL) and certain other B-cell malignancies and have been associated with accelerated disease progression and inferior responses to chemotherapy. Using in vitro and in vivo murine models and primary patient samples, we now show that NFKBIE-mutated CLL cells are selected by microenvironmental signals that activate the NF-κB pathway and induce alterations within the tumor microenvironment that can allow for immune escape, including expansion of CD8+ T-cells with an exhausted phenotype and increased PD-L1 expression on the malignant B-cells. Consistent with the latter observations, we find increased expression of exhaustion markers on T-cells from patients with NFKBIE-mutated CLL. In addition, we show that NFKBIE-mutated murine CLL cells display selective resistance to ibrutinib and report inferior outcomes to ibrutinib treatment in NFKBIE-mutated CLL patients. These findings suggest that NFKBIE mutations can contribute to CLL progression through multiple mechanisms, including a bidirectional crosstalk with the microenvironment and reduced sensitivity to BTK inhibitor treatment.

## Introduction

Chronic lymphocytic leukemia (CLL) is characterized by the progressive accumulation of autoreactive B lymphocytes driven to expand by B-cell receptor (BCR) and other microenvironmental signals and cooperating genetic lesions [1–4]. The genetic lesions that underlie CLL are heterogeneous and include copy number alterations and point mutations in over 200 putative CLL driver genes that cluster into several distinct biological pathways, including cell cycle regulation, DNA damage response, RNA processing, chromatin modification, and BCR, WNT, NOTCH and NF-kB signaling [5, 6].

The NF-κB family of transcription factors consists of homo- or heterodimers of the subunits p65, RelB, c-Rel, p50 and p52, which function as transcriptional activators or repressors of numerous genes that regulate cell proliferation, survival and differentiation [7, 8]. These transcription factors are maintained inactive in the cytoplasm by the IκB inhibitor proteins, including the typical inhibitors IκBα, IκBβ, and IκBε, the atypical inhibitors IκBζ, BCL-3, IκB-NS, and IκBη, and the precursor proteins p100 and p105 [9]. Activation of NF-κB transcription factors occurs in response to numerous microenvironmental signals and can involve the canonical or non-canonical pathway. The canonical pathway is activated by proinflammatory cytokines, Toll-like receptor (TLR) ligands or antigen-receptor stimulation and results in the phosphorylation and degradation of the typical IκB inhibitors, allowing for the release of the NF-κB transcription factors and their translocation from the cytoplasm into the nucleus. Activation of the non-canonical NF-κB pathway is induced by certain TNF superfamily members, such as CD40L and BAFF, and involves the phosphorylation and processing of p100 into p52 and subsequent nuclear translocation of p52/RelB heterodimers. However, substantial crosstalk exists between the two NF-κB signaling pathways, with NF-κB proteins from both pathways competing to form dimers [10, 11].

Mutations in the NFKBIE gene, which encodes the IκBε inhibitor, have been reported at frequencies ranging from 4% to 11% of untreated CLL patients and have been associated with shorter treatment-free survival and overall survival in patients treated with chemoimmunotherapy [12–16]. The NFKBIE mutations are typically truncating mutations and have been reported to result in loss of IκBε expression [13]. The most common mutation is a 4 bp frameshift deletion, which has also been detected in other B-cell malignancies, including diffuse large B-cell lymphoma, mantle cell lymphoma and at a particularly high frequency in primary mediastinal B-cell lymphoma [17].

In this study, we investigated the functional consequences of NFKBIE mutations using CRISPR/Cas9-edited murine models and primary patient samples. We show that NFKBIE mutations contribute to disease progression by increasing the growth rate of CLL cells stimulated with microenvironmental signals that activate NF-κB and by inducing alterations within the tumor microenvironment that can allow for immune evasion of the malignant B-cells. In addition, we show that NFKBIE mutations confer resistance to the BTK inhibitor ibrutinib in the murine models and are associated with inferior outcomes to ibrutinib treatment in CLL patients.

## Materials and methods

### CLL samples

Blood samples were collected from CLL patients after obtaining informed consent according to the Declaration of Helsinki. Study approval was obtained from the IRB of the Aviano Centro di Riferimento Oncologico. Analysis of NFKBIE mutations was done by next-generation sequencing in 249 CLL cases, including a cohort of 229 ibrutinib-treated cases reported elsewhere (Supplementary Table 1) [18].

### *CRISPR/Cas9-editing* of murine and human CLL cells

The Alt-R system (Integrated DNA Technologies) was used to introduce loss-of-function NFKBIE-mutations in previously established Eμ-TCL1-derived leukemia lines as described elsewhere [19, 20]. The Cas9 ribonucleoprotein complexes were generated by combining a predesigned NFKBIE crRNA (1.5 μM) with 1.5 μM ATTO550-labeled trans-activating crRNA, 0.75 μM recombinant Cas9 protein, and 1.5 μM Alt-R Cas9 electroporation enhancer in 5 μL duplex buffer and were then electroporated using the Amaxa Nucleofector II device and Z-001 program into 6 × 10^6^ leukemic cells resuspended in 100 μl Mouse B-cell Nucleofector solution (Lonza).

For CRISPR/Cas9 editing of human CLL cells, thawed cells were transfected with the Cas9 ribonucleoprotein complexes using the NEPA21 Super Electroporator (Nepagene) and transfection parameters listed in Supplementary Table 2. The transfected cells were then cultured for 72 h with 3T3-msCD40L fibroblasts, 5 ng/mL human IL-4 and 25 ng/mL of human IL-21 prior to additional 48 h culture in the presence of 3T3-CD40L fibroblasts, CpG ODN2006, or anti-IgM. Editing efficiency was evaluated by amplicon capillary electrophoresis on a 3500 Genetic Analyzer (Applied Biosystems) or by Sanger sequencing, as described elsewhere [19]. Cr-RNA and PCR primer sequences are provided in Supplementary Tables 3 and 4. Additional information is provided in the Supplementary Materials and Methods.

## Results

### NFKBIE-mutated murine CLL cells are positively selected by microenvironmental signals that activate the NF-kB pathway

To investigate how NFKBIE-mutations affect leukemia cell growth, we disrupted by CRISPR/Cas9 editing the NFKBIE gene in the murine TCL1-355-TKO cell line, which we recently established from the transgenic Eμ-TCL1 CLL model [19]. This cell line expresses an autoreactive BCR that binds to the apoptosis-associated autoantigen phosphatidylcholine (PtC) [21] and was generated by introducing biallelic loss-of-function mutations in TP53, CDKN2A and CDKN2B, which genes are co-affected in ~40% of Richter Syndrome tumors [22, 23]. The TCL1-355-TKO cells proliferate spontaneously in vitro but still remain BCR dependent, as evidenced by the rapid block of their proliferation in the presence of a BCR inhibitor and their selective loss in vitro and in vivo following genetic disruption of the IgM constant region gene [19]. In addition, these cells remain responsive to other putative microenvironmental signals, such as the TLR9 ligand CpG-DNA, which induces an increase in their proliferation rate following in vitro stimulation (Supplementary Fig. 1).

**Fig. 1:**
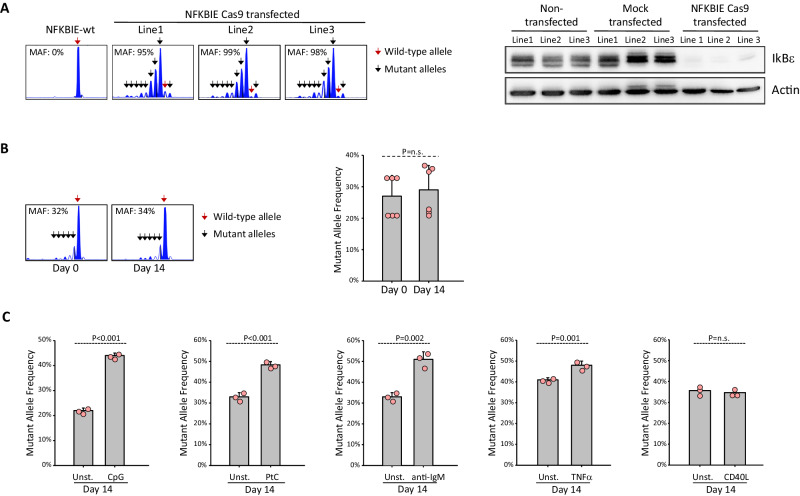
Murine TCL1-355-TKO cells with disrupted NFKBIE gene are positively selected in vitro by microenvironmental signals that activate the NF-kB pathway. **A** Generation of NFKBIE-ko TCL1-355-TKO cell lines. Left panel shows indel analysis by amplicon capillary electrophoresis of the three NFKBIE-ko TCL1-355-TKO cell lines that were generated in three independent experiments. Wild-type alleles are indicated by a red arrow; mutant alleles are indicated by a black arrow. Editing efficiency was determined by analyzing the NFKBIE mutant allele frequency (MAF). MAF was calculated by dividing the area of the mutated alleles with the total area of all amplified alleles (mutant + WT) detected in the amplicon capillary electrophoresis. Right panel shows immunoblotting analysis of IκBε protein expression in the three NFKBIE-ko TCL1-355-TKO lines. The same cells non-transfected or transfected with Cas9 without cr-RNA (mock transfected) were used as positive controls for IκBε protein expression. **B** TCL1-355-TKO NFKBIE-ko and NFKBIE-wt cells were mixed at a 1:2 or 1:4 ratio (*n* = 3 experiments per each condition) and cultured for 14 days at a cell density of 0.4–1.2 × 10^6^ cells/ml. Analysis of MAF was done at day 0 and day 14. Statistical analysis was done using *t* test. Error bars represent standard deviation. **C** Analysis of MAF at day 14 of mixed cultures of TCL1-355-TKO NFKBIE-ko and NFKBIE-wt cells stimulated with CpG-DNA (1 μM), PtC (0.5 mM), anti-IgM (20 μg/ml), TNF-α (20 μg/ml) or 3T3-msCD40L fibroblasts (1:20 ratio versus CLL cells). The ratio of NFKBIE-ko vs NFKBIE-wt cells was 1:4 in the CpG-stimulation experiment, 1:2 in the PtC, anti-IgM and TNF-α stimulation experiments and approximately 1:1 in the CD40L stimulation experiment, respectively. The cells were collected every 48 h, washed, resuspended in fresh medium and re-stimulated with the same concentration of the indicated stimuli (or the same cell ratio in the case of CD40L stimulation).

To evaluate the efficiency of the CRISPR/Cas9 editing, we analyzed cells obtained from three independent experiments by amplicon capillary electrophoresis and nucleotide sequencing of the CRISPR/Cas9-targeted region. Both approaches revealed an editing efficiency of close to 100% in the three lines that were generated (Fig. 1A, left panel, and Supplementary Fig. 2). Consistent with this finding, immunoblotting analysis showed almost complete absence of IκBε protein expression in each of the three TCL1-355-TKO NFKBIE-knockout (ko) lines (Fig. 1A, right panel). Loss of IκBε expression remained stable over time and no difference was observed in the growth rate of the NFKBIE-ko lines compared to their wild type (wt) counterparts (Supplementary Fig. 2).

To further investigate the effect of loss of IκBε expression on the growth of TCL1-355-TKO cells, we first conducted in vitro competition experiments with NFKBIE-ko and NFKBIE-wild type (wt) cells mixed at a 1:2 or 1:4 ratio. Analysis of the clonal composition after two weeks in culture revealed no change in the mutant allele frequency (MAF), further suggesting that IκBε loss does not affect the spontaneous in vitro growth of TCL1-355-TKO cells (Fig. 1B). However, repeated stimulation through the TLR, BCR or TNF receptor with CpG-DNA, anti-IgM, PtC or TNF-α, respectively, resulted in a significant increase in MAF, suggesting that loss of IκBε confers a growth advantage to TCL1-355-TKO cells when stimulated through the canonical NF-κB pathway (Fig. 1C). In contrast, no increase in MAF was noted following CD40L stimulation, which is involved in non-canonical NFκB signaling in murine B cells. The growth advantage of NFKBIE-ko cells in response to stimulation with the TLR ligands CpG and LPS was further validated in independent experiments with another TCL1 leukemia line with combined TP53, CDKN2A and CDKN2B disruption (Supplementary Fig. 3) [19].

**Fig. 2:**
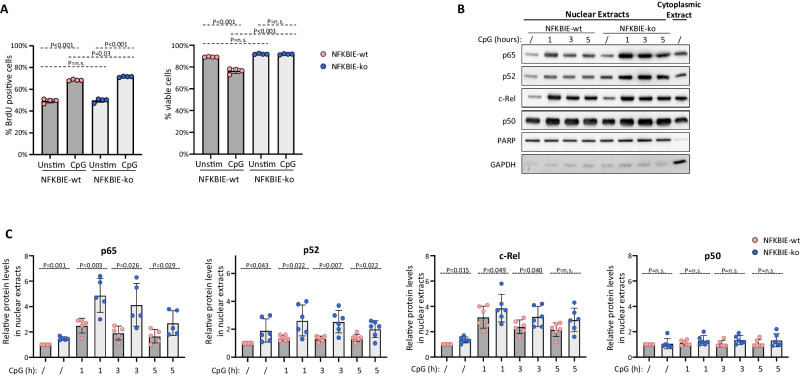
Impact of IκBε loss on the proliferation, survival and NF-κB pathway activation of Eμ-TCL1 CLL cells following CpG stimulation. **A** Analysis of cell proliferation by BrdU incorporation and cell viability by Annexin V/PI staining performed after 24 h culture with 1 μM CpG-ODN 1668. Cells were cultured for 6 h with BrdU prior to harvesting for flow cytometry analysis. Statistical analysis was done using One Way ANOVA with Tukey test for multiple comparisons. **B**, **C** Analysis of nuclear translocation of NF-κB transcription factors following CpG-DNA stimulation of NFKBIE-ko and NFKBIE-wt TCL1-355-TKO cells. Nuclear and cytoplasmic proteins were extracted before or after 1 h, 3 h and 5 h CpG-stimulation. One representative experiment is shown in **B** and a summary of 5 experiments for p65 and 6 for p52, c-Rel and p50 is shown in **C**. Statistical analysis was done with the paired *t* test.

To understand the mechanism through which IκBε loss provides a growth advantage to TCL1-355-TKO cells, we investigated the effects of CpG stimulation on the proliferation, survival and NF-κB pathway activation of NFKBIE-wt and NFKBIE-ko cells. An increase in both the percentage of proliferating and the percentage of apoptotic cells was observed after 24 h of CpG stimulation of NFKBIE-wt cells, suggesting that such stimulation increases the proliferation rate but also results in activation-induced apoptosis. In contrast, no increase in apoptosis was observed in NFKBIE-ko cells, which also showed a greater increase in the percentage of proliferating cells, suggesting that both increased resistance to activation-induced apoptosis and a greater capacity to respond to proliferative stimuli may underlie the growth advantage of these cells (Fig. 2A). Accordingly, immunoblotting analysis of nuclear and cytoplasmic protein extracts derived from NFKBIE-ko and NFKBIE-wt cells stimulated with CpG showed significantly greater nuclear translocation of the NF-κB subunits p65, c-Rel and p52 in NFKBIE-ko cells, suggesting that loss of IκBε provides a growth advantage by increasing the transcriptional activity of these NF-κB subunits (Fig. 2B, C).

### NFKBIE-mutated human CLL cells are positively selected by CpG and CD40L stimulation

**Fig. 3:**
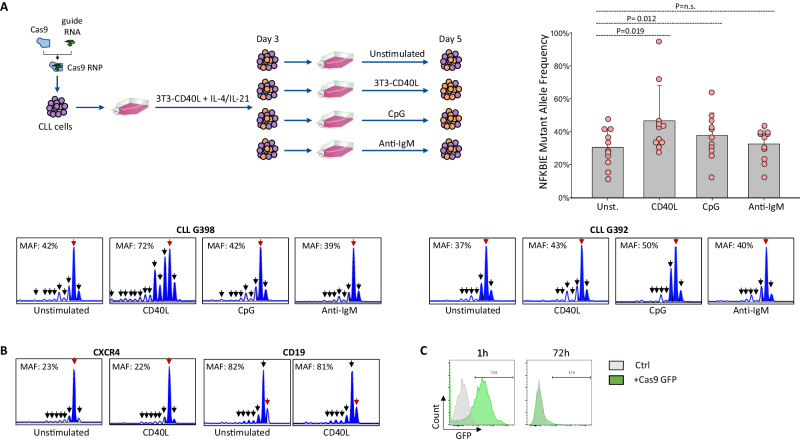
Human CLL cells with loss-of-function NFKBIE mutations are positively selected in vitro by microenvironmental signals that activate the NF-κB pathway. **A** Analysis of effects of microenvironmental signals involved in NF-κB pathway activation on the growth of NFKBIE-mutated primary human CLL cells. NFKBIE mutations were introduced in primary leukemic cells from 11 CLL patients with wild type NFKBIE by CRISPR/Cas9 editing. The cells were cultured for 3 days with 3T3-CD40L fibroblasts (1:10 ratio) +IL-4/IL-21 before being split and cultured for additional 48 h alone or in the presence of 3T3-CD40L fibroblasts, CpG ODN2006 (1 μM), or anti-IgM (20 μg/ml). NFKBIE mutant allele frequency was determined at day 5 of culture. The upper left panel provides a schematic representation of the experimental approach. The graph in the upper right panel represents the summary of the results with the 11 different CLL samples. Statistical analysis was done using the Wilcoxon signed-rank test. The results of two representative samples (G398 and G392) are shown in the bottom panels. **B** Analysis of effects of CD40L-stimulation on the growth of CXCR4- or CD19-edited primary human CLL cells. The same experimental approach was used as in part A, except for the use of guide RNAs targeting human CXCR4 or CD19 instead of NFKBIE. Capillary plots of the targeted region of the CXCR4 and CD19 genes from one representative sample out of 3 analyzed are shown. Wild-type alleles are indicated by a red arrow; mutant alleles are indicated by a black arrow. **C** Flow cytometry analysis of one CLL sample transfected with GFP-labeled Cas9 protein. Analysis was done after 1 h and after 72 h from the transfection.

Although the TCL1 transgenic mouse model is widely accepted as a relevant tool for the study of microenvironmental interactions in CLL, there are notable differences with respect to the human disease [24]. These differences particularly refer to the requirement for proliferation and survival signals from T cells, which are essential for the growth of human CLL cells but dispensable for the growth of murine TCL1 CLL cells in in vivo transplantation models [25, 26]. In addition, CD40L, which is predominantly expressed on activated CD4+ T cells, has been reported to activate both the canonical and noncanonical pathway in human CLL cells, in contrast to murine B-cells where it selectively activates the noncanonical pathway [27, 28]. For these reasons, we investigated whether human CLL cells with loss-of-function NFKBIE mutations will be positively selected following stimulation with CD40L, CpG or anti-IgM. To introduce NFKBIE mutations, primary CLL cells from 11 patients were transfected with Cas9 ribonucleoprotein complexes targeting human NFKBIE and were then cultured for 3 days with 3T3-CD40L fibroblasts + IL-4/IL-21 to induce their proliferation, which is required to achieve efficient Cas9 editing [29]. The edited cells were then split and cultured for additional 2 days in the absence of any stimuli or in the presence of 3T3-CD40L fibroblasts, CpG, or anti-IgM. A significant increase in NFKBIE MAF was observed in CLL cells cultured with 3T3-CD40L fibroblasts or with CpG compared to unstimulated cells, whereas no difference was observed in CLL cells stimulated with anti-IgM (Fig. 3A). To confirm that the observed increase in MAF is because of positive selection of NFKBIE-mutated cells and not because of continuous Cas9 editing in CD40L- or CpG-stimulated CLL cells, we used the same approach to investigate the outcome of loss-of-function mutations in the CXCR4 or CD19 gene. These genes were chosen as controls because loss of their expression cannot provide a growth advantage and therefore an increase in CXCR4 or CD19 MAF would only occur in case of continuous Cas9 editing. As shown in Fig. 3B, no difference in CXCR4 or CD19 MAF was observed between CLL cells cultured in the presence or absence of 3T3-CD40L fibroblasts. Moreover, time-course experiments with a Cas9-GFP protein transfected in human CLL cells showed complete disappearance of the signal after 72 h coculture on 3T3-CD40L fibroblasts, further suggesting that the observed increase of NFKBIE MAF in cells stimulated with CD40L or CpG was because of positive selection of cells with such mutations (Fig. 3C).

### NFKBIE-mutated cells are positively selected in vivo

**Fig. 4:**
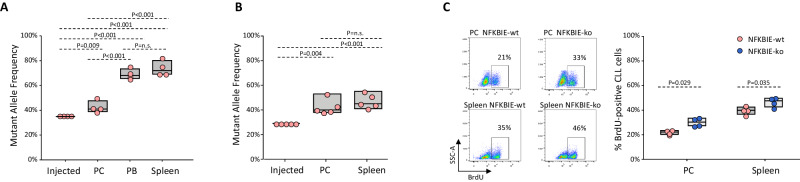
Murine CLL cells with disrupted NFKBIE are positively selected by the tumor microenvironment in vivo. **A** TCL1-355-TKO NFKBIE-ko and NFKBIE-wt cells were mixed at a 1:2 ratio and injected in 4 C57BL/6 mice (2 × 10^7^ cells/mouse). Cells were isolated from the PC, peripheral blood (PB) and spleen of the injected C57BL/6 mice 3 weeks after adoptive transfer and NFKBIE MAF was determined in purified leukemia cells by amplicon capillary electrophoresis of the targeted region. Statistical analysis was done using One-way Repeated Measures ANOVA with the Holm-Sidak test for multiple comparisons. **B** NFKBIE MAF in injected leukemia cells and cells isolated from the PC and spleen of NSG mice 3 weeks after transplantation. The TCL1-355-TKO NFKBIE-ko and NFKBIE-wt cells were mixed at a 1:2.5 ratio and injected in 5 NSG mice (2 × 10^7^ cells/mouse). Statistical analysis was done as above. **C** In vivo BrdU-incorporation analysis of TCL1-355 TKO NFKBIE-wt or NFKBIE-ko cells isolated from PC and spleen of NSG mice (n = 4/group). BrdU was injected intraperitoneally 21 days after adoptive transfer. Leukemic cells were collected after 12 h, purified by negative selection using a B cell isolation kit, and analyzed by flow cytometry. Flow charts show the analysis of one NFKBIE-wt and one NFKBIE-ko sample, summary of the data is shown in the graph. Statistical analysis was performed using the Mann–Whitney Rank Sum Test.

We next investigated whether NFKBIE-mutated cells will also be selected in vivo. To address this question, we transplanted by intraperitoneal injection mixtures of NFKBIE-ko and NFKBIE-wt TCL1-355-TKO cells into syngeneic recipient mice and investigated 3 weeks later the NFKBIE MAF of cells isolated from the peritoneal cavity (PC), blood and spleen of the transplanted mice. We observed significant enrichment of NFKBIE-mutant alleles in the recovered cells from all three compartments compared to the injected cells (Fig. 4A). Interestingly, leukemia cells isolated from the spleen and blood showed a significantly greater increase in MAF compared to leukemia cells isolated from PC, implying the involvement of different selection mechanisms in different compartments. The difference in MAF between the two compartments was lost when the same cells were transplanted into immunodeficient NSG mice, indicating a possible role for the immune system in the selection of the NFKBIE-ko cells (Fig. 4B). However, in vivo BrdU labeling of NSG mice that had received either NFKBIE-wt or NFKBIE-ko cells showed a higher percentage of proliferating leukemia cells in the latter cohort, suggesting that the growth advantage of the NFKBIE-ko cells was at least in part a consequence of a greater capacity to respond to proliferative microenvironmental signals (Fig. 4C).

### NFKBIE-mutated cells induce transcriptional changes in the immune microenvironment

To further investigate the mechanism(s) through which loss of IκBε confers a growth advantage to the malignant B-cells, we performed RNA-seq analysis of tumors with NFKBIE-ko and NFKBIE-wt TCL1-355-TKO cells. For this purpose, the NFKBIE-ko and NFKBIE-wt TCL1-355-TKO cells were first separately propagated in two C57BL/6 mice and then transplanted via intraperitoneal injection into two groups of C57BL/6 recipient mice (5 mice/group). The leukemia cells were recovered 10 days later from the spleen and PC of the transplanted mice and 4 samples from each group were subjected to RNA sequencing. Importantly, TCL1-355-TKO cells were not purified in these experiments to allow for the analysis of non-leukemic elements from the tumor microenvironment, which ranged from 5 to 15% of the mononuclear cells in each individual sample. In parallel, to determine the impact of the NFKBIE mutation on the transcriptional profile of unstimulated cells, we analyzed NFKBIE-ko and NFKBIE-wt TCL1-355-TKO cells that were isolated from the spleens of 3 different mice and separately cultured for two weeks prior to RNA isolation.

**Fig. 5:**
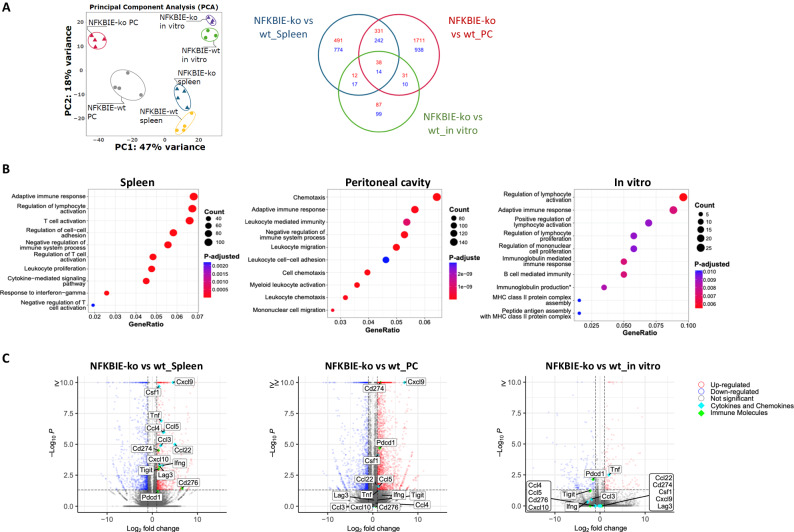
Transcriptome analysis of NFKBIE-wild type and NFKBIE-mutated tumors. **A** Principal Component Analysis of the transcript expression counts (log2 vst-method normalized counts) for the 22 samples showing the first versus the second PC. PC1 shows 47% variance and PC2 18%. Each dot represents a NFKBIE-wt sample, each triangle is a NFKBIE-ko sample. Right panel shows Venn diagram of DETs. Numbers in each circle represent the number of DETs between the different comparisons while the ones overlapping are for mutual DETs. Red numbers represent upregulated DETs while blue numbers represent downregulated DETs. **B** Top ten Gene Ontology terms based on the Gene Ratio (x-axis), which is the ratio between the transcripts of the set found to be enriched for that specific term and the total transcripts in the set. In each row, the dots represent the enriched biological terms. The size of each dot is proportional to the number of the transcripts enriched in the term, while the color of the dots is the p-adjusted value, spanning from red to blue. **C** Volcano plots of significantly upregulated transcripts (red) and significantly down-regulated transcripts (blue) based on *p* < 0.05 and fold change ≥ 1 or ≤−1, respectively; black dots represent non-differentially expressed transcripts. Transcripts with a *P* value equal to or lower than 10^−10^ were clustered together.

Principal Component Analysis separated the NFKBIE-ko from the NFKBIE-wt samples as well as the in vitro and the two different in vivo conditions, further indicating differences between the selection mechanisms that operate in the different anatomical compartments (Fig. 5A). Functional annotation of the differentially expressed transcripts (DETs) across the different anatomical compartments showed that most of the top significantly overrepresented biological processes in the spleens were related to T-cell activation and cytokine signaling, whereas the most differentially expressed processes in the PC and the in vitro condition were related to chemotaxis/leukocyte migration and lymphocyte activation, respectively (Fig. 5B). A more detailed inspection of the DETs in the spleen revealed enrichment of various cytokines and chemokines involved in the recruitment of T-cells and macrophages, such as Ccl3, Ccl4, Ccl5, Ccl22, Cxcl9, Cxcl10, Csf1, Tnfα and Ifnγ, as well as inhibitory immune checkpoint molecules, such as CD274 (Pd-l1), Tigit, Lag3, Pdcd1 (Pd-1) and CD276 in mice that received NFKBIE-ko cells (Fig. 5C). Except for Tnfα, none of these molecules were enriched in NFKBIE-ko cells compared to NFKBIE-wt cells under the in vitro condition, suggesting that they are induced by signals from the microenvironment. Consistent with this possibility, only some of these molecules were differentially expressed in the PC.

To understand which of the DETs were expressed by the leukemic and which by the non-leukemic cells, we performed RNAseq analysis of leukemia cells purified from the spleens of each of the 10 mice from the above experiment. This analysis combined with the previous analysis identified 1852 DETs that originated from the leukemic cells and 813 DETs that originated from the non-leukemic cells (Supplementary Fig. 4). Among the differentially expressed immune regulatory molecules, the chemokines Ccl3, Ccl4, Ccl5, Ccl22, Cxcl9 and Cxcl10, the cytokines Csf1 and Tnfα and the inhibitory immune checkpoint ligand CD274 (Pd-l1) were found to originate from the leukemic cells, whereas Ifnγ, Tigit, Lag3 and Pdcd1 (Pd-1) were found to originate from the non-leukemic cells.

### NFKBIE-mutated CLL cells induce expression of exhaustion markers on T-cells

**Fig. 6:**
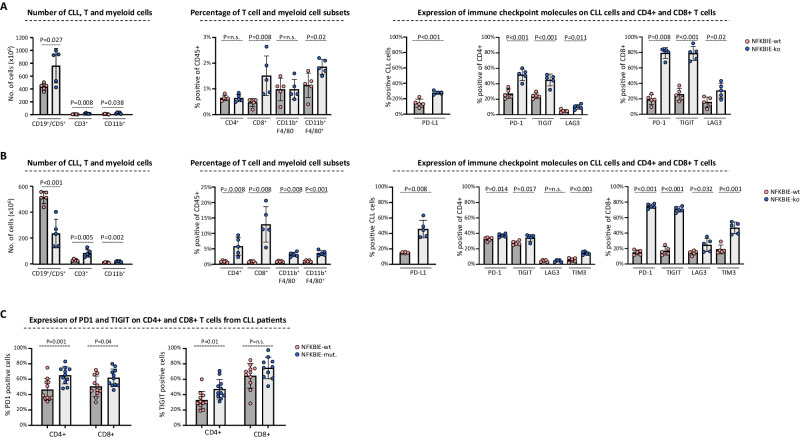
Immune composition analysis of NFKBIE-wt and NFKBIE-ko tumors. **A** Analysis of total number and percentage of different immune cells and expression of immune checkpoint molecules in spleens of mice injected with NFKBIE-wt or NFKBIE-ko leukemia cells (*n* = 5/group). The investigated populations included CLL cells (CD19^+^CD5^+^), T cells (CD3^+^CD4^+^ and CD3^+^CD8^+^), granulocytes (CD11b^+^F4/80^−^), and monocytes/macrophages (CD11b^+^F4/80^+^) gated on viable CD45^+^ cells. Spleen samples from the experiment in Fig. 5 were used for this analysis. **B** Analysis of immune cell number and composition and immune checkpoint expression in spleens from a separate experiment with mice that were injected with 3 × 10^7^ NFKBIE-wt or 1 × 10^7^ NFKBIE-ko leukemia cells. **C** Expression of PD-1 and TIGIT on CD4^+^ and CD8^+^ T cells from patients with NFKBIE-wt and NFKBIE-mutated CLL (MAF > 20%).

The findings from the RNA-seq analysis further indicated that immune evasion mechanisms may be involved in the selection of NFKBIE-ko cells in the spleens of the transplanted mice. To further explore this possibility, we analyzed the different cell populations in the spleens of mice transplanted with NFKBIE-ko or NFKBIE-wt CLL cells using flow cytometry (Fig. 6A). In addition to a greater number of leukemic cells, we observed a significantly greater number of T-cells and myeloid cells in the spleens of mice with NFKBIE-ko tumors, consistent with the greater expression of chemoattractants for these cells by NFKBIE-ko cells. The increase in the number of T-cells and myeloid cells was mainly caused by the greater recruitment of CD8+ T-cells and CD11b^+^F4/80^+^ monocytes. In addition, we detected increased expression of PD-L1 on the NFKBIE-ko leukemia cells as well as increased expression of PD-1, TIGIT and LAG3 on CD4+ and CD8+ T-cells from the spleens of mice with NFKBIE-ko tumors, consistent with the exhausted phenotype predicted by the RNAseq analysis.

To exclude the possibility that the T-cell compartment changes were a consequence of a greater tumor burden due to the proliferative advantage of the NFKBIE-ko cells, which could eventually result in more frequent stimulation and more rapid exhaustion of the infiltrating T-cells, we repeated the transplantation experiment using a 3 times lower number of NFKBIE-ko compared to NFKBIE-wt cells (Fig. 6B). Analysis of spleens of mice isolated 13 days after transplantation confirmed the previously observed greater T-cell recruitment and increased expression of the exhaustion markers PD-1, TIGIT, LAG3 and TIM3, despite the reduced tumor burden in mice that received NFKBIE-ko cells. Additional T-cell subset analysis revealed an expansion of CD8+ effector memory T-cells (CD44^+^CD62L^−^) in mice that had received NFKBIE-ko TCL1-355-TKO cells, which T-cell population also showed the highest percentage of cells expressing PD-1 and TIGIT (Supplementary Fig. 5).

To investigate whether similar changes occur in the human setting, we analyzed the expression of PD-1 and TIGIT on CD4+ and CD8+ T-cells in peripheral blood samples from 11 NFKBIE-mutated (MAF > 0.2) and 11 NFKBIE-wt CLL patients. Consistent with the murine data, significantly higher expression was observed for PD-1 on CD4+ and CD8+ T-cells and for TIGIT on CD4+ T-cells from the NFKBIE-mutated cases (Fig. 6C).

### NFKBIE-mutations selectively affect the response to ibrutinib treatment

**Fig. 7:**
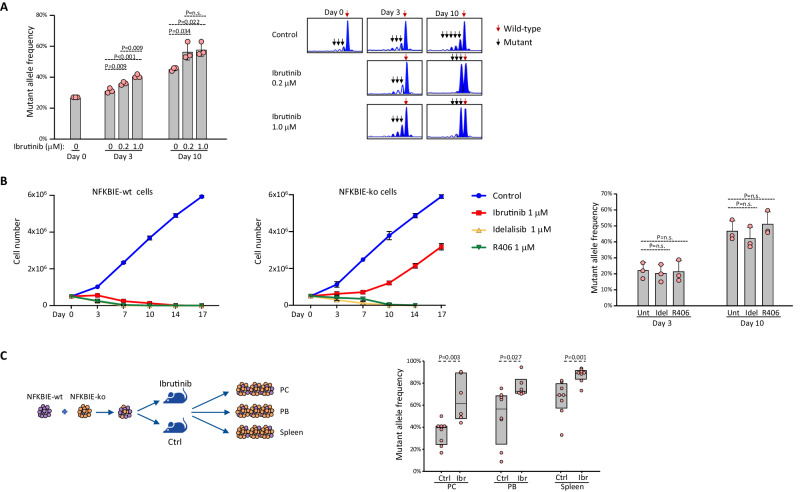
Analysis of BCR inhibitor sensitivity of NFKBIE-wt and NFKBIE-ko CLL cells. **A** Changes in NFKBIE MAF during ibrutinib treatment in vitro. NFKBIE-ko and NFKBIE-wt TCL1-355 TKO cells were mixed at a 1:3 ratio and cultured in the presence or absence of ibrutinib (0.2 or 1.0 µM). DMSO was present at a concentration of 0.5% in both the ibrutinib-treatment and the control condition. DNA was isolated at the indicated time points for MAF analysis. Summary of the data is shown in the left panel and analysis of one representative sample from each condition in the right panels. Statistical analysis was done using One Way ANOVA with Tukey test for multiple comparisons. **B** Growth curve analysis of NFKBIE-wt and NFKBIE-ko CLL cells in culture with the indicated inhibitors. Each data point represents an average of 3 independent experiments. Graph in right panel shows changes in NFKBIE MAF during R406 and idelalisib treatment in vitro. NFKBIE knockout and wild type TCL1 leukemia cells were mixed as in A and cultured in the presence or absence of 1.0 μM idelalisib or R406. DMSO was used at a concentration of 0.5% in all investigated conditions. Statistical analysis was done using One Way ANOVA with Holm-Sidak test for multiple comparisons. **C** Changes in NFKBIE MAF during ibrutinib treatment in vivo. NFKBIE-knockout and NFKBIE-wild type leukemia cells were mixed at a 1:3 ratio and injected in 16 wild-type mice. Treatment was started three days later (8 mice treated with ibrutinib and 8 with vehicle control) and lasted for 2 weeks. The MAF at the end of treatment for the control (Ctrl) or ibrutinib (Ibr)-treated group is shown in the graph. The PB and PC samples from 2 ibrutinib-treated mice were lost during processing and were not available for analysis. Statistical analysis was performed with the *t* test (PC and PB) and Mann-Whitney rank sum test (spleen).

Because mutations in other NF-κB pathway genes, including BIRC3, TRAF2, TRAF3, MAP3K14 and CARD11, have been associated with ibrutinib resistance in mantle cell lymphoma [30, 31], we next investigated whether mutations in NFKBIE could also affect the response to ibrutinib treatment. For this purpose, we first tested the in vitro proliferation rate of NFKBIE-ko and NFKBIE-wt TCL1-355-TKO cells in the presence of two ibrutinib concentrations, one below and one above the maximal plasma concentration achievable in patients (0.2 µM and 1.0 µM, respectively). At both concentrations, ibrutinib inhibited the proliferation of NFKBIE-ko cells to a significantly lesser extent compared to NFKBIE-wt cells (Supplementary Fig. 6). Consistent with this finding, in vitro competition experiments with mixtures of NFKBIE-ko and NFKBIE-wt cells showed positive selection of the mutated cells in the presence of ibrutinib, as evidenced by the significant increase in MAF on days 3 and 10 of culture (Fig. 7A). Interestingly, in this experiment the NFKBIE-ko cells were positively selected even in the absence of ibrutinib, albeit to a lesser degree, which contrasted with the previous findings showing no growth advantage for unstimulated NFKBIE-ko cells in vitro. Since previous studies had reported that the solvent in these experiments, DMSO, can inhibit NF-κB activity [32, 33], we investigated the growth of the NFKBIE-ko and NFKBIE-wt cells in the presence or absence of DMSO. Time-course analysis showed an increase over time of NFKBIE MAF in cells cultured with but not in those cultured without DMSO (Supplementary Fig. 7). To determine the mechanism responsible for this selection, we investigated the effect of DMSO on the survival and proliferation of NFKBIE-ko and NFKBIE-wt TCL1-355 TKO cells. DMSO induced apoptosis and inhibited the proliferation of NFKBIE-wt cells to a significantly greater extent compared to NFKBIE-ko cells, suggesting that the NFKBIE-ko cells were selected because of greater resistance to the cytotoxic and growth inhibitory effects of DMSO (Supplementary Fig. 8).

In contrast to ibrutinib, the presence of NFKBIE mutations did not affect sensitivity to treatment with the PI3K inhibitor idelalisib or the SYK inhibitor R406, as both drugs effectively inhibited the proliferation of NFKBIE-ko cells in vitro (Fig. 7B). Moreover, in vitro competition experiments showed no change in the proportion of NFKBIE-ko cells in the presence of idelalisib or R406 over a period of 10 days in culture, further suggesting that NFKBIE mutations confer selective resistance to ibrutinib treatment.

The greater resistance of NFKBIE-mutated cells to ibrutinib was further validated by an in vivo competition experiment. A mixture of NFKBIE-ko and NFKBIE-wt TCL1-355-TKO cells was inoculated intraperitoneally into syngeneic C57BL/6 mice which were then treated with ibrutinib or vehicle control. Analysis of leukemia cells isolated from PC, PB and spleen after 2 weeks of treatment showed a significantly greater increase in NFKBIE MAF in mice treated with ibrutinib compared to control mice in all investigated compartments (Fig. 7C).

**Fig. 8:**
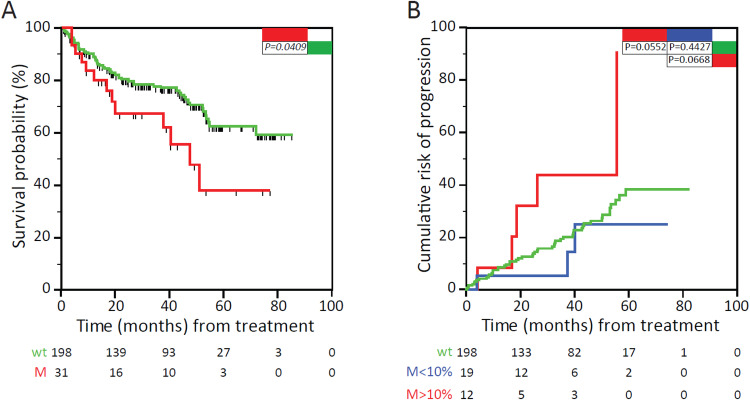
Survival and progression analyses from initiation of ibrutinib treatment in CLL patients segregated based on NFKBIE mutations. **A** Overall survival calculated from initiation of ibrutinib therapy of CLL patients with wild type NFKBIE (green curve) vs mutated NFKBIE (red curve). **B** Cumulative risk of progression under ibrutinib treatment of CLL patients with wild type NFKBIE (green curve) vs high NFKBIE MAF (M > 10%, red curve) vs low NFKBIE MAF (M < 10%, blue curve).

To investigate whether NFKBIE mutations can also affect the response to ibrutinib treatment in the clinical setting, we analyzed overall survival and cumulative risk of progression in a cohort of 229 ibrutinib-treated CLL patients for whom follow-up clinical data were available (Supplementary Table 1). NFKBIE mutations were detected by targeted NGS in pretreatment samples of 13.5% of these patients (31/229), among whom 5.2% (12 cases) had a MAF of >10% and 8.3% (19 cases) had a MAF of <10%. A significantly reduced overall survival calculated from the beginning of ibrutinib treatment was documented for the NFKBIE-mutated compared to the NFKBIE-wt patients (*P* = 0.0409; Fig. 8A), whereas a higher cumulative risk of progression on ibrutinib treatment was observed for NFKBIE-mutated patients with a MAF greater than 10% (*P* = 0.0552; Fig. 8B).

## Discussion

Mutations in the NFKBIE gene are among the most common genetic lesions that target the NF-κB pathway in CLL, but how they contribute to CLL pathogenesis and progression is still poorly understood. The data presented in this study show that such mutations can provide a growth advantage to the leukemic cells by at least two different mechanisms, including increased capacity to respond to various growth and survival signals from the microenvironment and induction of changes in the microenvironment that can contribute to immune evasion of the malignant B-cells.

Previous studies have reported that IκBε-deficient mouse B1 and marginal zone B-cells hyperproliferate in response to stimuli that activate the NF-κB pathway, such as the TLR agonists CpG and LPS or anti-IgM [34, 35]. Consistent with these findings, we show that leukemic murine B-cells with IκBε deficiency display a higher proliferation rate and greater apoptosis resistance when exposed to such stimuli. More importantly, we show that primary human CLL cells with NFKBIE mutations are preferentially selected following stimulation with CpG or CD40L, which stimuli are considered potential drivers of CLL cell proliferation [25, 36, 37].

Interestingly, we also observed a greater selection of NFKBIE-mutated murine leukemia cells in immunocompetent compared to immunodeficient mice, suggesting that the presence of NFKBIE mutations may impact the anti-tumor immune response. CLL is a malignancy that is characterized by substantial immune system alterations, including expansion of dysfunctional CD8+ T-cells that display increased expression of various exhaustion markers, including PD-1, TIM3, LAG3, CD160, CD244 and TIGIT [38–45]. Many of these changes were observed in the spleens of mice that received NFKBIE-mutated cells, including an increase in the number of CD8+ T-cells and increased expression of PD-1, TIGIT, LAG3 and TIM3. In addition, we observed greater expression of PD-1 and TIGIT on T-cells from CLL patients with NFKBIE-mutated leukemia, further suggesting that NFKBIE mutations in CLL cells can promote T-cell exhaustion and contribute to escape from immune surveillance.

Genetic lesions in other CLL or lymphoma driver genes have also been reported to induce changes in the immune microenvironment that could potentially lead to immune escape of the malignant B-cells. In particular, inactivating mutations in CREBBP and overexpression of activated NOTCH1 or NOTCH2 have been reported to provide potential immune escape by downregulating MHC class II and upregulating PD-L1 expression on the malignant B-cells, thus reducing their immunogenicity [46–48]. In our model, introduction of NFKBIE mutations also resulted in increased expression of PD-L1 on the malignant B-cells but did not result in reduced MHC class II gene expression (Supplementary Fig. 9), suggesting that NFKBIE mutations induce changes in the immune microenvironment through a different mechanism. Although the exact mechanism is currently unknown, it is plausible that such changes could be mediated by some of the multitude of immunoregulatory cytokines and chemokines that were overexpressed by the NFKBIE-mutated CLL cells, many of which are well-established direct NF-κB targets.

Another important finding of our study is the association between NFKBIE mutations and reduced sensitivity to ibrutinib treatment. Although mutations in BTK and/or PLCG2 have been established as the dominant mechanism of ibrutinib resistance in CLL, in many patients such mutations are present in only a minor subclone at the time of progression, suggesting that additional genetic lesions may contribute to ibrutinib resistance [49–52]. Other genetic lesions that have been implicated in ibrutinib resistance include mutations in NOTCH1 and biallelic lesions in TP53, which have been associated with shorter progression-free survival and overall survival when compared to patients without such genetic lesions or with single TP53 abnormalities [18, 53, 54]. Inferior outcomes to ibrutinib treatment were also observed in the NFKBIE-mutated patients from our study, which included the same patients investigated in the study of Bomben et al. [18]. It is noteworthy that these inferior outcomes were detected despite the limited number of patients with NFKBIE mutations and the fact that a high proportion of cases in the control group had concomitant TP53 mutation and deletion. Inferior responses to ibrutinib treatment were also seen in our murine models, showing that in vitro ibrutinib inhibits the proliferation of NFKBIE-mutated cells to a lesser extent compared to NFKBIE-wt cells, and that in vivo NFKBIE-mutated cells are positively selected during ibrutinib treatment. Importantly, the presence of NFKBIE mutations did not result in increased resistance to inhibitors of the kinases PI3Kδ and SYK, which are positioned proximally to BTK and therefore, in addition to NF-κB, can more profoundly inhibit other pathways downstream of the BCR that also provide growth and survival signals [55].

In conclusion, in this study we have generated novel human and murine CLL and Richter Syndrome model systems to show that malignant cells with truncation mutations in NFKBIE are selected by growth signals provided by the tumor microenvironment, that such cells can impact the anti-tumor immune response and that such mutations can impart resistance to ibrutinib therapy. These model systems can be interrogated in future studies for mechanistic insight into why malignant cells with truncation mutations in NFKBIE are resistant to ibrutinib and how such cells cause changes in the immune microenvironment that can contribute to immune escape.

### Supplementary information

Supplementary Figures, Tables and Materials and Methods

## Data Availability

All data from this manuscript are available in the published article and its online supplemental material or in the GEO repository under accession number GSE231799.
