# Supplementary material for: NFKBIE mutations are selected by the tumor microenvironment and contribute to immune escape in chronic lymphocytic leukemia
Source: Leukemia. 2024 Mar 15;38(7):1511–21. doi: 10.1038/s41375-024-02224-8 (PMC11216988; doi:10.1038/s41375-024-02224-8)
Supplement: Supplementary file 1 — Supplementary Figures, Tables and Materials and Methods [file 41375_2024_2224_MOESM1_ESM.pdf]

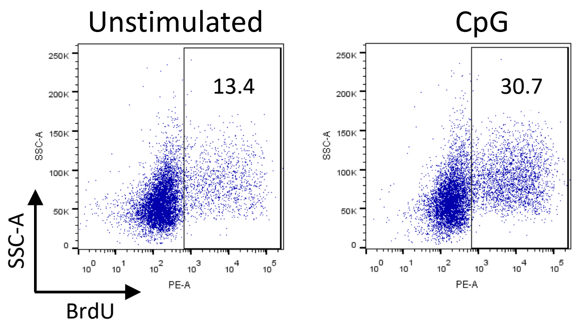

Supplementary Figure 1. Analysis of BrdU incorporation of unstimulated or CpG-stimulated TCL1-355-TKO leukemia cells. Cells were stimulated for 24 hours with 1 μM CpG-ODN 1668 and then cultured for additional 3 hours with 10 μM BrdU prior to harvesting for flow cytometry analysis.

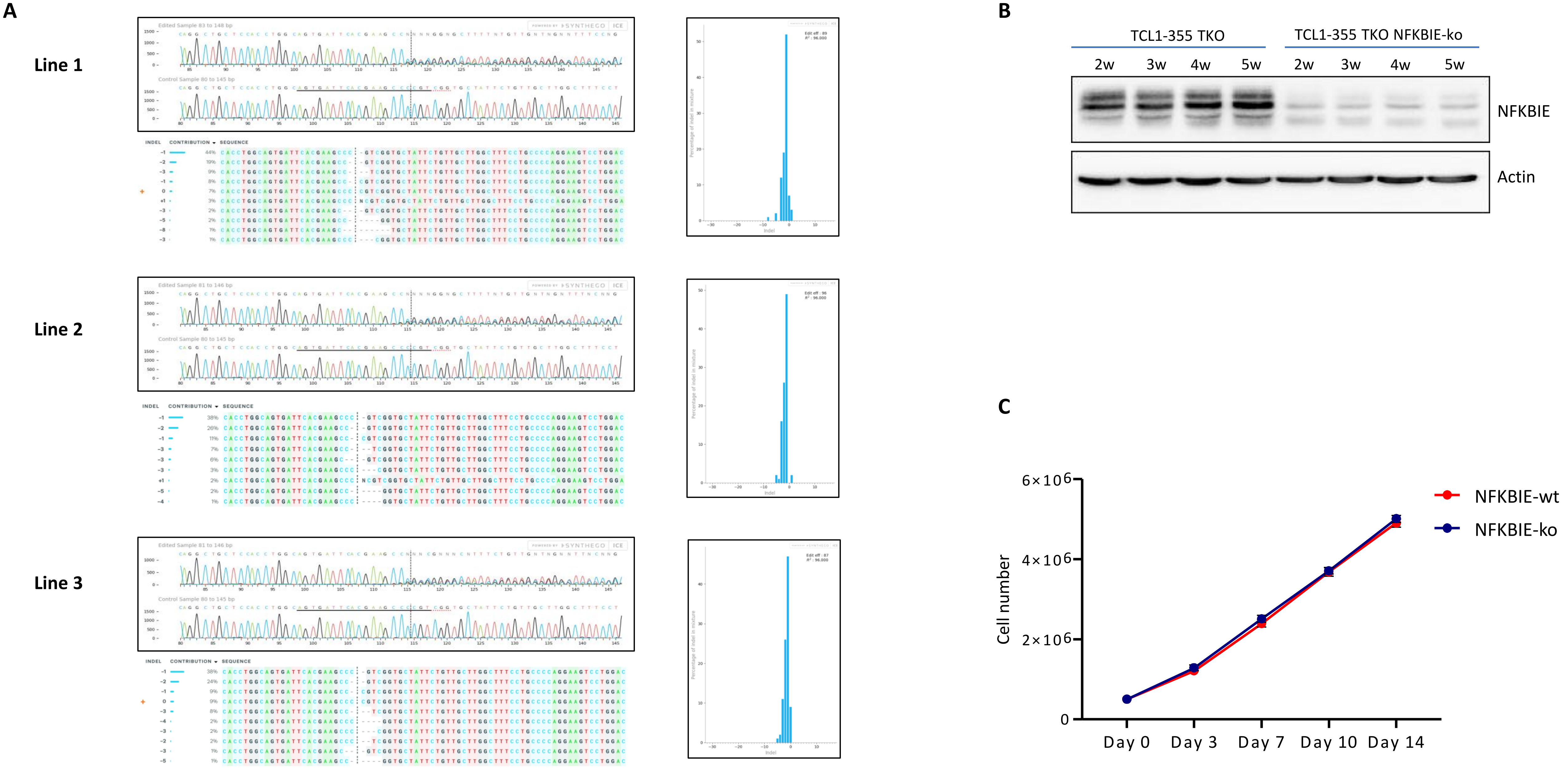

Supplementary Figure 2. Analysis of NFKBIE editing efficiency and growth characteristics of the TCL1-355 TKO NFKBIE-wild type (wt) and NFKBIE-knockout (ko) cells. (A) Nucleotide sequences of the NFKBIE Cas-9 targeted region of the 3 independently established NFKBIE-ko lines and their wt counterparts are shown in the left panels. Analysis of the percentage of NFKBIE indels using the ICE software tool is shown in the right panels. Wild type allele corresponds to position 0. B) Immunoblotting analysis of NFKBIE expression on samples of line 3 collected at weekly (w) intervals. C) Growth curves of TCL1-355 TKO NFKBIE-wt and NFKBIE-ko (line 2) cells.

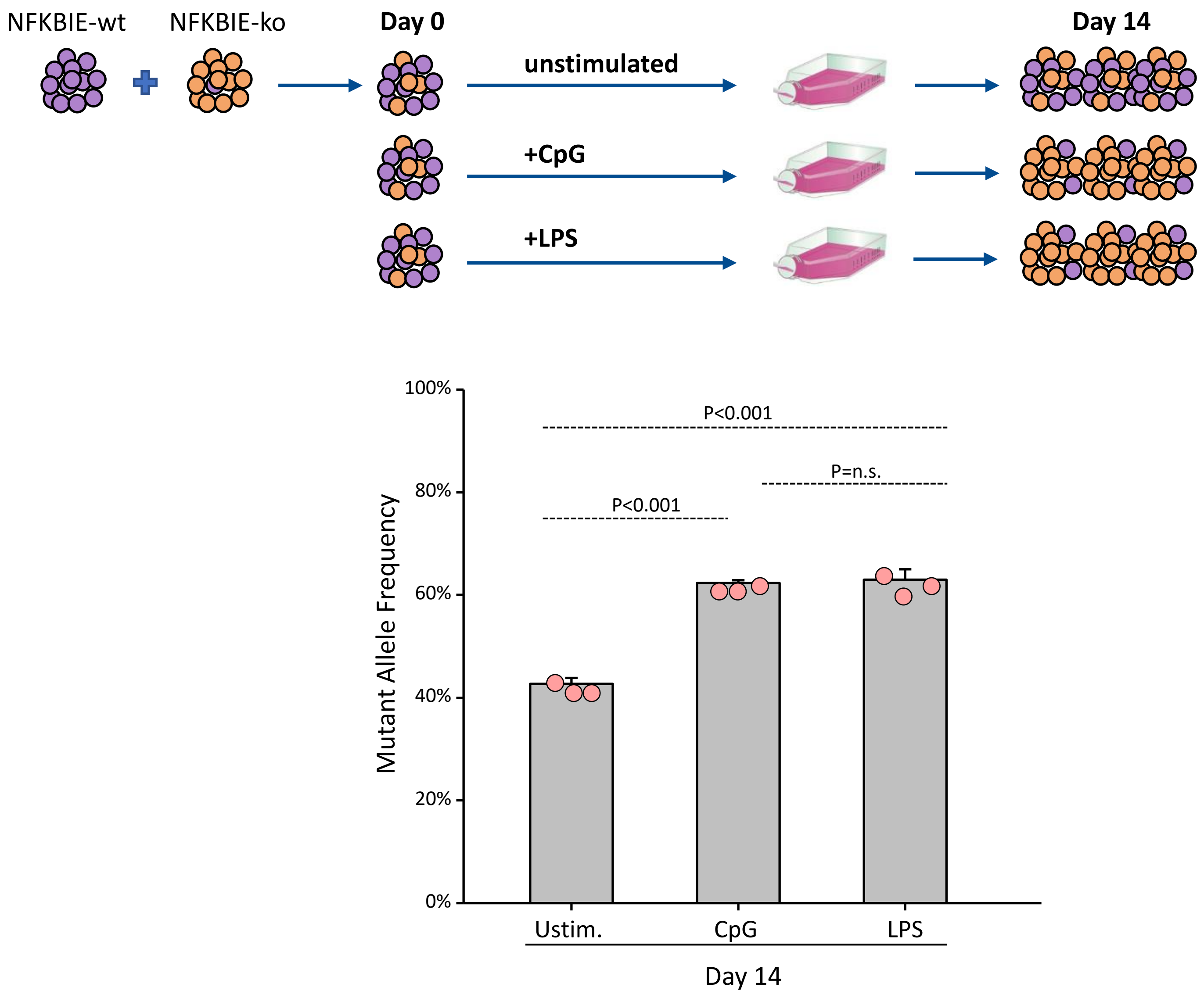

Supplementary Figure 3. Analysis of Mutant Allele Frequency during in vitro culture of mixtures of NFKBIE-ko and NFKBIE-wt TCL1-699 TKO cells in presence or absence of CpG-DNA (1 µM/mL) or lipopolysaccharides (LPS) (5 µg/mL). Statistical analysis was done using One Way ANOVA with Tukey test for multiple comparisons.

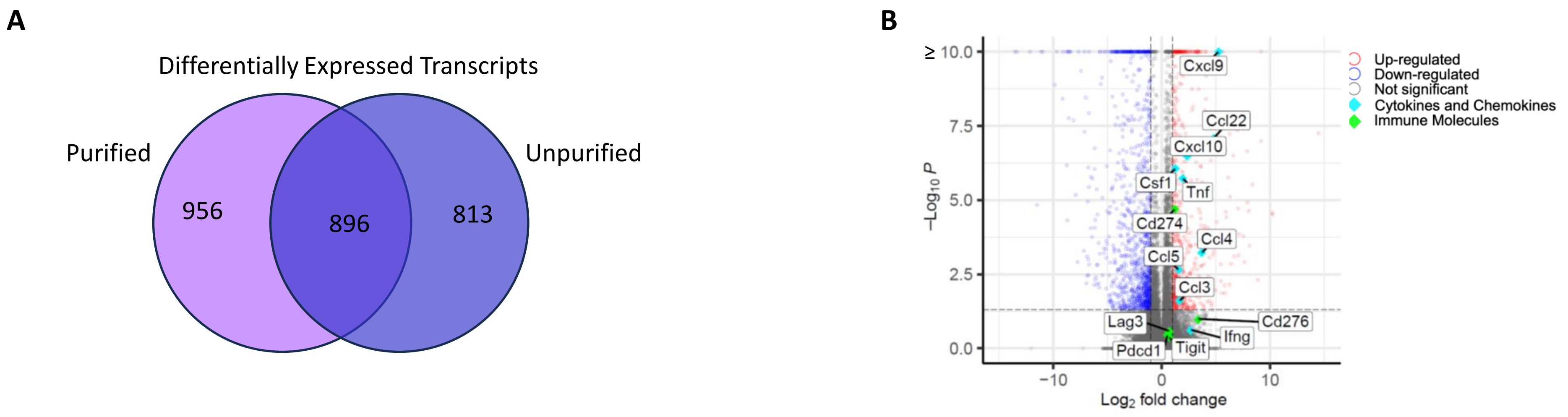

Supplementary Figure 4. Transcriptome analysis of CD19-purified leukemia cells from NFKBIE-wt and NFKBIE-ko tumors. (A) Differentially expressed transcripts detected in unpurified or CD19-purified splenic samples. (B) Volcano plots of significantly upregulated transcripts (red) and significantly down-regulated transcripts (blue) differentially expressed between NFKBIE-ko and NFKBIE-wt TCL1-355 TKO cells from CD19-purified splenic samples.

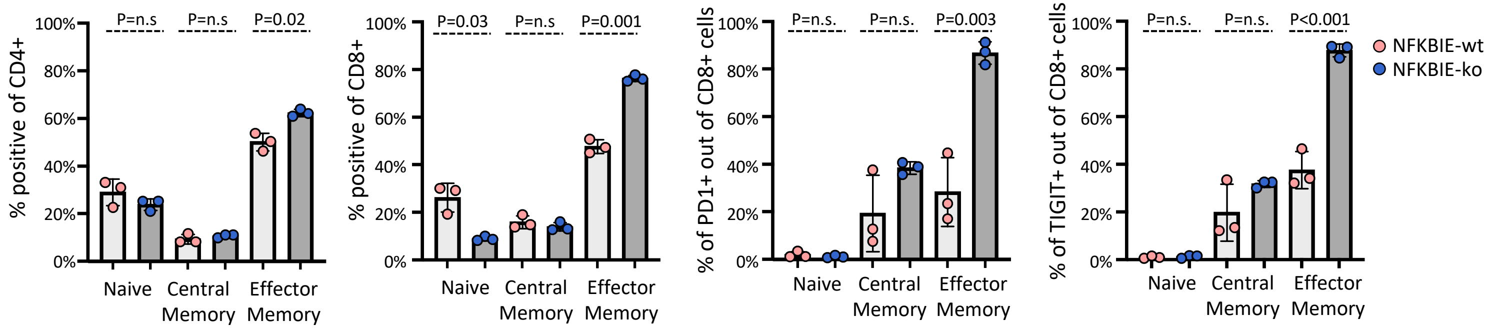

Supplementary Figure 5. Analysis of naive (CD44-CD62L+), central memory (CD44+CD62L+) and effector memory (CD44+CD62L-) T cells in spleens of mice injected with NFKBIE-wt or NFKBIE-ko leukemia cells.

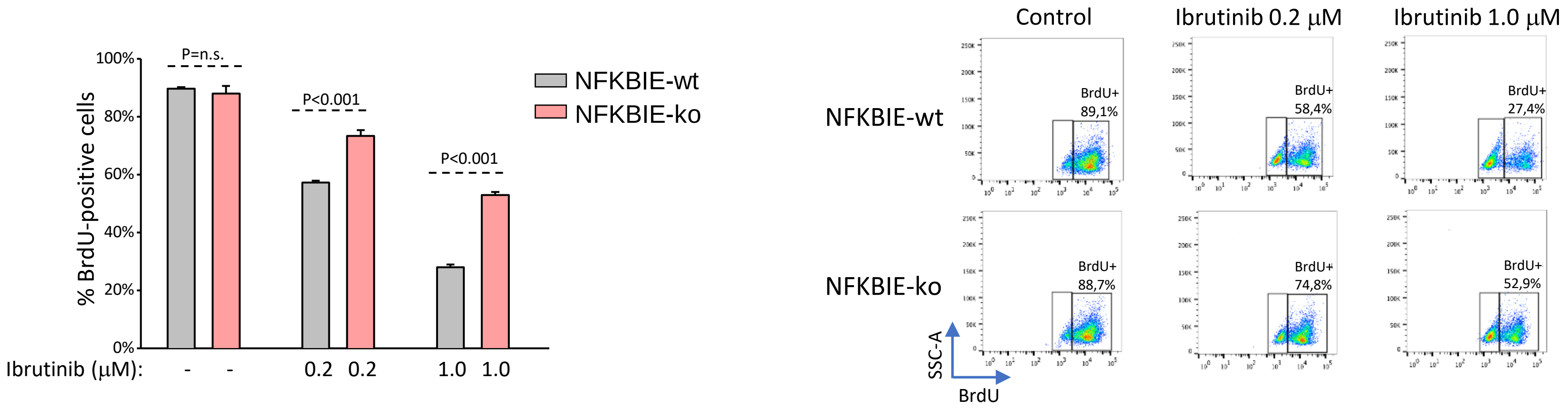

Supplementary Figure 6. Analysis of BrdU incorporation in TCL1-355-TKO NFKBIE-ko and NFKBIE-wt cells treated with ibrutinib. Graph shows summary of results from 3 independent experiments, one of these experiments is shown in the right panels. Analysis was done after 48 hours of ibrutinib treatment, BrdU was present in the culture medium during the last 12 hours. Significance of differences with respect to untreated cells was evaluated with the paired t test.

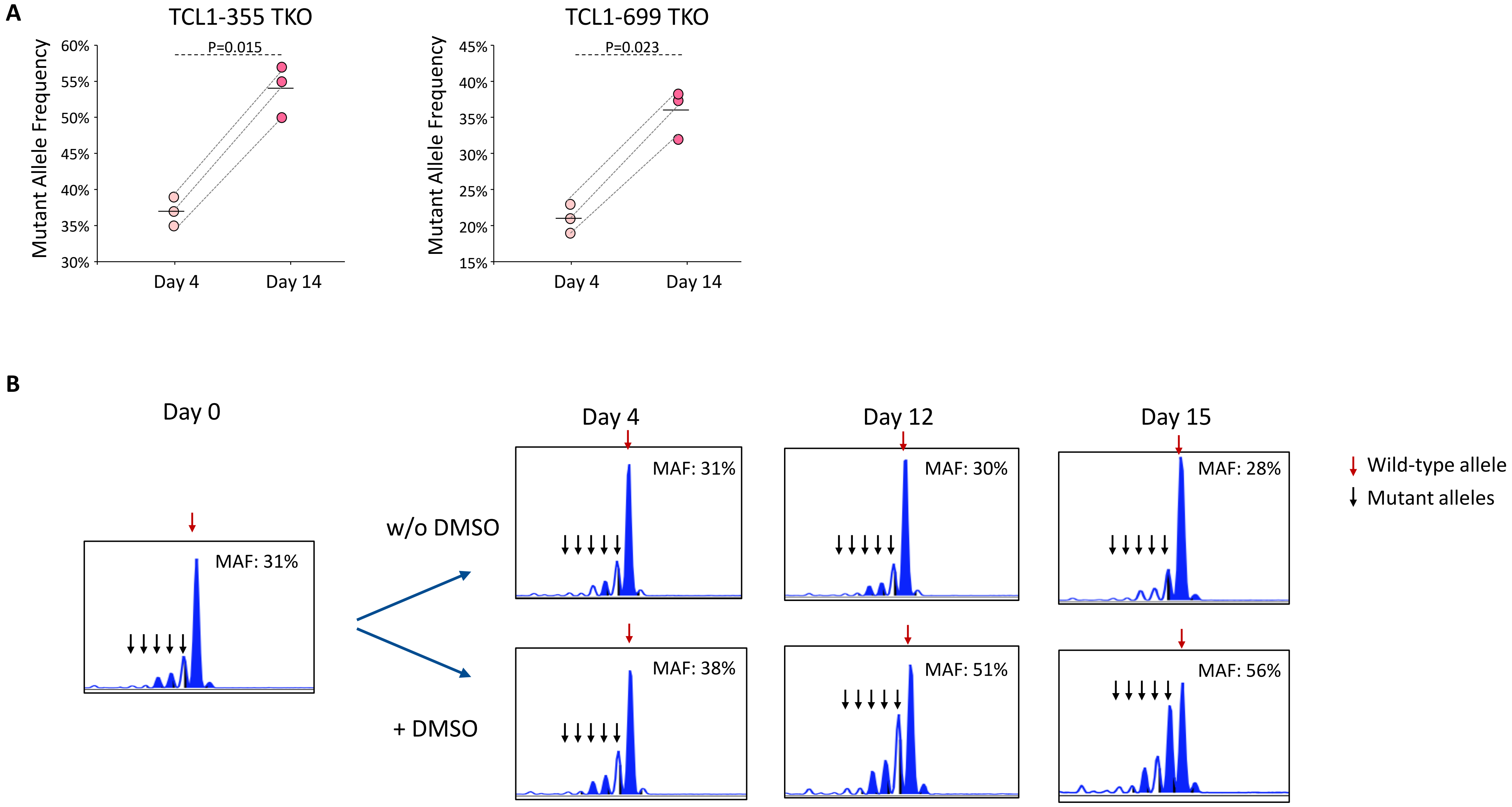

Supplementary Figure 7. Analysis of Mutant Allele Frequency during in vitro culture of mixtures of NFKBIE-ko and NFKBIE-wt TCL1-355 TKO or TCL1-699 TKO cells in presence or absence of DMSO. (A) NFKBIE-ko and NFKBIE-wt TCL1-355 TKO or TCL1-699 TKO cells were mixed at different ratios, split and cultured in the presence of 0.5% DMSO for 14 days. A significant increase in NFKBIE MAF was observed at day 14 compared to day 4. (B) NFKBIE-ko and NFKBIE-wt TCL1-355 TKO cells were mixed at a 1:2 ratio, split and cultured in the presence or absence of 0.5% DMSO for 15 days. MAF was determined by PCR and amplicon capillary electrophoresis on day 0, 4, 12 and 15. Shown is one out of three experiments with identical results.

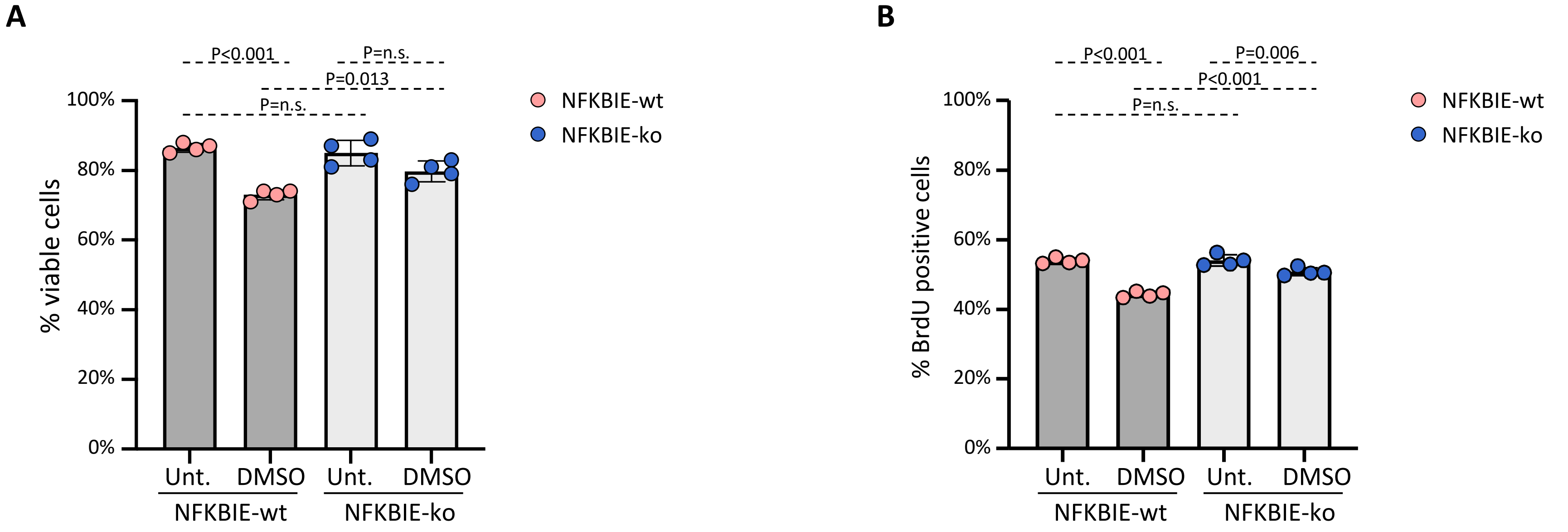

Supplementary Figure 8. Effect of DMSO on the survival and proliferation of NFKBIE-ko and NFKBIE-wt TCL1-355 TKO cells. (A) Analysis of cell viability by Annexin V/PI staining performed after 24 hours in culture with or without 0.5% DMSO. B) Analysis of cell proliferation by BrdU incorporation performed after 44 hours in culture with or without 0.5% DMSO. Cells were cultured for the last 6 hours with BrdU prior to harvesting for flow cytometry analysis. Statistical analysis was done using One Way ANOVA with Tukey test for multiple comparisons.

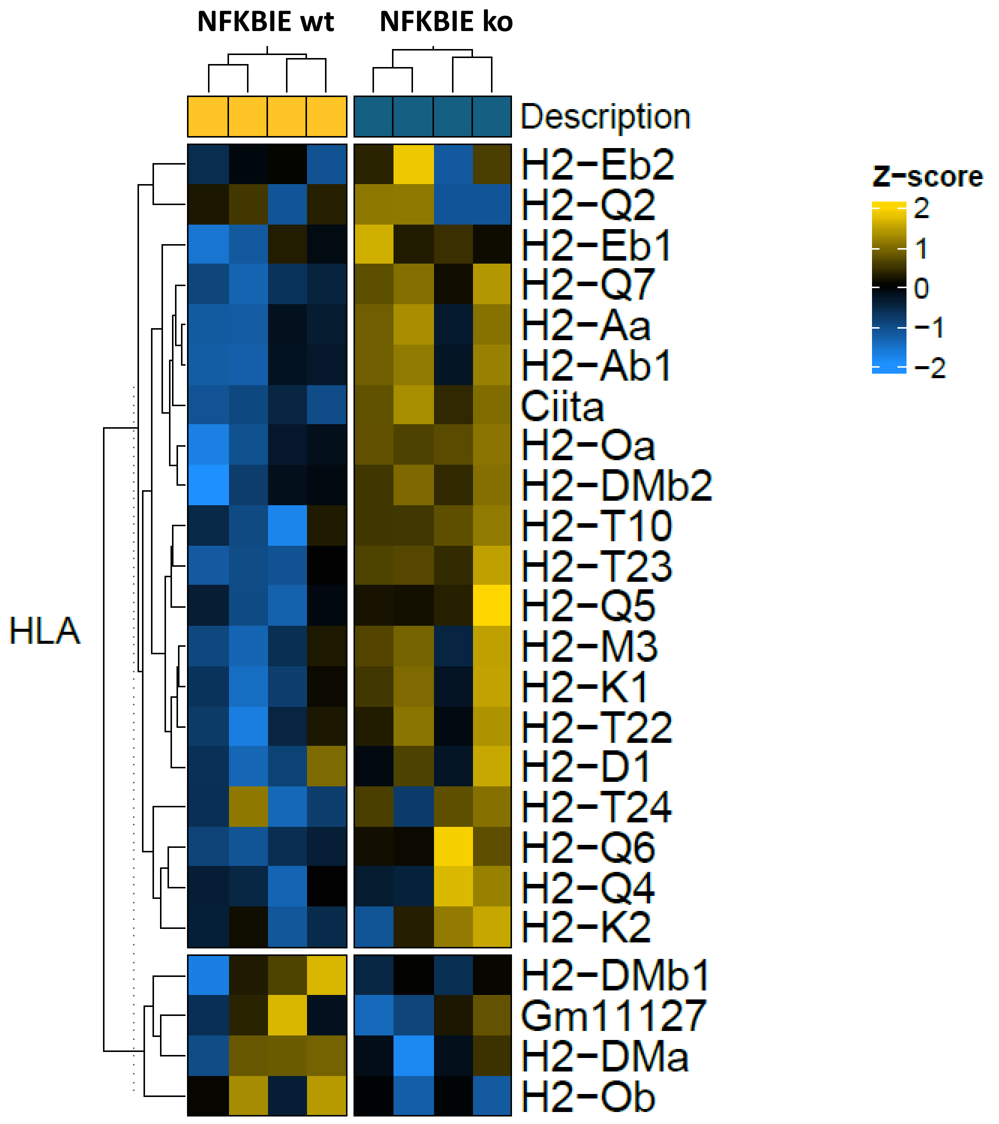

Supplementary Figure 9. Heatmap of Z-score of HLA and Ciita canonical transcripts across spleen NFKBIE-wt and NFKBIE-ko samples (4 each), each grouping together with its own sample type (gold: NFKBIE wt, blue: NFKBIE ko). Z-score color varies from blue (negative values) to yellow (positive values). “Average” was used as clustering method and “correlation” for clustering distance of both rows and columns.

Supplementary Table I. Primary CLL cohort and *NFKBIE* mutation features

Sample_ID	Protein Change	Mutation Type	Variant allele frequency	high-VAF / low-VAF†	Coding‡
BE-GL	wt		0	wt	
Br-Gi	wt		0	wt	
Ce-Fu	wt		0	wt	
CO-NC	wt		0	wt	
CRO406	Y254Sfs*13	deletion	1.8	low-VAF	c.759_762del,p.Y254Sfs*13
CT148	wt		0	wt	
CT151	wt		0	wt	
CT163	wt		0	wt	
CT168	wt		0	wt	
CT186	wt		0	wt	
CT219	wt		0	wt	
CT255	wt		0	wt	
CT265	wt		0	wt	
CT280	wt		0	wt	
CT282	wt		0	wt	
CT291	Y254Sfs*13	deletion	2.5	low-VAF	c.759_762del,p.Y254Sfs*13
CT297	wt		0	wt	
CT304	wt		0	wt	
CT308	wt		0	wt	
CT310	wt		0	wt	
CT338	wt		0	wt	
CT366	wt		0	wt	
CT373	Y254Sfs*13	deletion	6.7	low-VAF	c.759_762del,p.Y254Sfs*13
CT38	wt		0	wt	
CT387	Y254Sfs*13	deletion	13.0	high-VAF	c.759_762del,p.Y254Sfs*13
CT398	wt		0	wt	
CT399	wt		0	wt	
CT403	wt		0	wt	
CT407	wt		0	wt	
CT412	Y254Sfs*13	deletion	7.7	low-VAF	c.759_762del,p.Y254Sfs*13
CT413	wt		0	wt	
CT426	wt		0	wt	
CT431	wt		0	wt	
CT436	wt		0	wt	
CT439	wt		0	wt	
CT440	wt		0	wt	
CT447	wt		0	wt	
CT453	wt		0	wt	
CT455	wt		0	wt	
CT60	wt		0	wt	
CT63	wt		0	wt	

CT92	wt		0	wt	
Da-MC	wt		0	wt	
El-En	wt		0	wt	
Gh-Di	wt		0	wt	
Li-Giu	wt		0	wt	
Lo-Ma	wt		0	wt	
Me-MG	wt		0	wt	
MODENA_1	wt		0	wt	
MODENA_11	wt		0	wt	
MODENA_12	wt		0	wt	
MODENA_13	wt		0	wt	
MODENA_14	wt		0	wt	
MODENA_15	wt		0	wt	
MODENA_16	wt		0	wt	
MODENA_17	wt		0	wt	
MODENA_18	wt		0	wt	
MODENA_2	wt		0	wt	
MODENA_3	wt		0	wt	
MODENA_4	wt		0	wt	
MODENA_6	wt		0	wt	
MODENA_7	wt		0	wt	
MODENA_8	wt		0	wt	
MODENA_9	wt		0	wt	
Pi-Gi	wt		0	wt	
RM113	wt		0	wt	
RM120	wt		0	wt	
RM121	wt		0	wt	
RM127	wt		0	wt	
RM13	wt		0	wt	
RM152	wt		0	wt	
RM187	Y254Sfs*13	deletion	4.5	low-VAF	c.759_762del,p.Y254Sfs*13
RM224	wt		0	wt	
RM28	wt		0	wt	
RM292	wt		0	wt	
RM295	wt		0	wt	
RM309	wt		0	wt	
RM312	wt		0	wt	
RM333	Y254Sfs*13	deletion	6.6	low-VAF	c.759_762del,p.Y254Sfs*13
RM394	wt		0	wt	
RM400	wt		0	wt	
RM422	wt		0	wt	
RM425	wt		0	wt	
RM442	wt		0	wt	
RM443	wt		0	wt	
RM459	Y254Sfs*13	deletion	9.9	low-VAF	c.759_762del,p.Y254Sfs*13
RM49	wt		0	wt	
RM499	wt		0	wt	

RM5	wt		0	wt	
RM531	wt		0	wt	
RM544	wt		0	wt	
RM576	wt		0	wt	
RM597	wt		0	wt	
RM6	wt		0	wt	
RM629	wt		0	wt	
RM632	wt		0	wt	
RM634	wt		0	wt	
RM653	Y254Sfs*13	deletion	3.2	low-VAF	c.759_762del,p.Y254Sfs*13
RM655	wt		0	wt	
RM664	wt		0	wt	
RM678	wt		0	wt	
RM684	wt		0	wt	
RM699	wt		0	wt	
RM709	wt		0	wt	
RM725	wt		0	wt	
RM731	wt		0	wt	
RM739	wt		0	wt	
RM76	wt		0	wt	
RM767	wt		0	wt	
RM770	Y254*	nonsense	61.1	high-VAF	c.762C>A,p.Y254*
RM770	Y254Sfs*13	deletion	4.3	low-VAF	c.759_762del,p.Y254Sfs*13
RM781	wt		0	wt	
RMGEM113	wt		0	wt	
RMGEM118	Y254Sfs*13	deletion	21.1	high-VAF	c.759_762del,p.Y254Sfs*13
RMGEM128	wt		0	wt	
RMGEM142	wt		0	wt	
RMGEM15	wt		0	wt	
RMGEM162	Y254Sfs*13	deletion	53.2	high-VAF	c.759_762del,p.Y254Sfs*13
RMGEM18	wt		0	wt	
RMGEM19	wt		0	wt	
RMGEM36	wt		0	wt	
RMGEM38	wt		0	wt	
RMGEM40	wt		0	wt	
RMGEM49	wt		0	wt	
RMGEM52	Y254Sfs*13	deletion	1.5	low-VAF	c.759_762del,p.Y254Sfs*13
RMGEM54	wt		0	wt	
RMGEM57	wt		0	wt	
RMGEM6	wt		0	wt	
RMGEM8	wt		0	wt	
RMGEM97	wt		0	wt	
RMPTV13	wt		0	wt	
RMPTV133	wt		0	wt	
RMPTV137	wt		0	wt	
RMPTV140	wt		0	wt	

RMPTV144	wt		0	wt	
RMPTV162	wt		0	wt	
RMPTV164	wt		0	wt	
RMPTV199	wt		0	wt	
RMPTV201	wt		0	wt	
RMPTV234	Y254Sfs*13	deletion	4.1	low-VAF	c.759_762del,p.Y254Sfs*13
RMPTV234	T261R	missense	3.2	low-VAF	c.782C>G,p.T261R
RMPTV241	wt		0	wt	
RMPTV261	wt		0	wt	
RMPTV277	wt		0	wt	
RMPTV293	wt		0	wt	
RMPTV312	wt		0	wt	
RMPTV320	wt		0	wt	
RMPTV322	wt		0	wt	
RMPTV324	wt		0	wt	
RMPTV325	Y254Sfs*13	deletion	1.7	low-VAF	c.759_762del,p.Y254Sfs*13
RMPTV33	wt		0	wt	
RMPTV331	wt		0	wt	
RMPTV341	wt		0	wt	
RMPTV343	wt		0	wt	
RMPTV353	wt		0	wt	
RMPTV354	wt		0	wt	
RMPTV366	wt		0	wt	
RMPTV368	Y254Sfs*13	deletion	1.8	low-VAF	c.759_762del,p.Y254Sfs*13
RMPTV376	wt		0	wt	
RMPTV42	Y254Sfs*13	deletion	2.4	low-VAF	c.759_762del,p.Y254Sfs*13
RMPTV441	wt		0	wt	
RMPTV472	wt		0	wt	
RMPTV51	wt		0	wt	
RMPTV53	wt		0	wt	
RMPTV8	wt		0	wt	
RMSA15	wt		0	wt	
RMSA16	wt		0	wt	
RMSA18	Y254Sfs*13	deletion	4.8	low-VAF	c.759_762del,p.Y254Sfs*13
RMSA24	wt		0	wt	
RMSA25	wt		0	wt	
RMSA30	wt		0	wt	
RMSA31	Y254*	nonsense	22.2	high-VAF	c.761dup,p.Y254*
RMSA35	wt		0	wt	
RMSA37	wt		0	wt	
RMSA41	wt		0	wt	
RMSA45	Y254*	nonsense	13.3	high-VAF	c.761dup,p.Y254*
RMSA47	wt		0	wt	
RMSA48	wt		0	wt	
RMSA51	wt		0	wt	

RMSA52	Y254Sfs*13	deletion	4.7	low-VAF	c.759_762del,p.Y254Sfs*13
RMSA53	wt		0	wt	
RMSA54	wt		0	wt	
RMSA58	wt		0	wt	
RMSA61	wt		0	wt	
RMSA64	wt		0	wt	
RMSA66	wt		0	wt	
RMSA67	Y254Sfs*13	deletion	19.7	high-VAF	c.759_762del,p.Y254Sfs*13
RMSA70	wt		0	wt	
RMSA9	wt		0	wt	
Sm-Es	wt		0	wt	
Ti-Ma	wt		0	wt	
TS134	wt		0	wt	
TS143	wt		0	wt	
TS161	wt		0	wt	
TS166	wt		0	wt	
TS208	wt		0	wt	
TS235	wt		0	wt	
TS242	wt		0	wt	
TS280	wt		0	wt	
TS290	wt		0	wt	
TS31	Y254Sfs*13	deletion	25.9	high-VAF	c.759_762del,p.Y254Sfs*13
TS33	wt		0	wt	
TS368	wt		0	wt	
TS375	wt		0	wt	
TS88	wt		0	wt	
UD101	wt		0	wt	
UD104	wt		0	wt	
UD115	wt		0	wt	
UD126	wt		0	wt	
UD129	wt		0	wt	
UD13	Y254Sfs*13	deletion	1.6	low-VAF	c.759_762del,p.Y254Sfs*13
UD141	Y254Sfs*13	deletion	50.7	high-VAF	c.759_762del,p.Y254Sfs*13
UD15	wt		0	wt	
UD150	Y254Sfs*13	deletion	1.4	low-VAF	c.759_762del,p.Y254Sfs*13
UD156	wt		0	wt	
UD161	wt		0	wt	
UD162	wt		0	wt	
UD173	Q248*	nonsense	3.2	low-VAF	c.742C>T,p.Q248*
UD173	Y254Sfs*13	deletion	2.3	low-VAF	c.759_762del,p.Y254fs*13
UD18	wt		0	wt	
UD185	wt		0	wt	
UD203	wt		0	wt	
UD216	wt		0	wt	

UD22	wt		0	wt	
UD30	Y254Sfs*13	deletion	7.0	low-VAF	c.759_762del,p.Y254Sfs*13
UD31	wt		0	wt	
UD4	wt		0	wt	
UD46	Y254Sfs*13	deletion	44.9	high-VAF	c.759_762del,p.Y254Sfs*13
UD65	Y254Sfs*13	deletion	66.8	high-VAF	c.759_762del,p.Y254Sfs*13
UD65	Y254*	nonsense	14.3	high-VAF	c.761dup,p.Y254*
UD74	Y254Sfs*13	deletion	49.7	high-VAF	c.759_762del,p.Y254Sfs*13
UD76	wt		0	wt	
UD85	wt		0	wt	
UD66	I268*	nonsense	12.45	high-VAF	c.800_801dup,p.I268X
RM419	wt		0		0
CT401	wt		0		0
RMPTV370	wt		0		0
RM719	wt		0		0
RM778	wt		0		0
CT82	wt		0		0
CT575	wt		0		0
UD12	wt		0		0
CT129	wt		0		0
CT544	wt		0		0
CT213	Y254Sfs*13	deletion	26.26	high-VAF	c.759_762del,p.Y254Sfs*13
RMPTV177	Y254Sfs*13	deletion	44.94	high-VAF	c.759_762del,p.Y254Sfs*13
CT58	Y254Sfs*13	deletion	35.07	high-VAF	c.759_762del,p.Y254Sfs*13
RMCA2	Y254Sfs*13	deletion	12.66	high-VAF	c.759_762del,p.Y254Sfs*13
CT27	Y254Sfs*13	deletion	55.87	high-VAF	c.759_762del,p.Y254Sfs*13
CS16	Y254Sfs*13	deletion	1	low-VAF	c.759_762del,p.Y254Sfs*13
RM843	Y254Sfs*13	deletion	0.6	low-VAF	c.759_762del,p.Y254Sfs*13
RMPTV361	Y254Sfs*13	deletion	4	low-VAF	c.759_762del,p.Y254Sfs*13

† high-VAF, $\geq$10.0% VAF; low-VAF, <10.0% VAF;

‡ According to Human Genome Variation Society (HGVS) nomenclature. https://www.mutalyzer.nl/;

For cases with multiple mutations the mutation with the highest VAF is highlighted (grey cell fill color).

Supplementary Table 2. Electroporation parameters used for transfection of human CLL cells with the NEPA21 Super Electroporator

No. of cells	Volume of electroporation medium*	Parameters	Poring pulse	Transfer pulse
$6x10^6$	100 µl	Voltage (V)	275	20
		Pulse length (ms)	1	50
		Pulse interval (ms)	50	50
		Number of pulses	4	5
		Decay rate (%)	10	40
		Polarity	+	+/-

*Opti-MEM Reduced Serum Medium was used as electroporation medium

Supplementary Table 3. Nucleotide sequences of cr-RNAs used in this study.

Name of the Gene	(5’-3’) cr-RNA sequences
NFKBIE (Mus musculus)	AGUGAUUCACGAAGCCCCGUGUUUUAGAGCUAUGCU
NFKBIE (Homo sapiens)	ACUCAAUGCCAGAGUCGUACGUUUUAGAGCUAUGCU
CXCR4 (Homo sapiens)	GAAGCAUGACGGACAAGUACGUUUUAGAGCUAUGCU
CD19 (Homo sapiens)	CGAGGAACCUCUAGUGGUGAGUUUUAGAGCUAUGCU

Supplementary Table 4. Sequences of primers used for PCR amplification of Cas9-targeted regions.

Name of the Gene	(5’-3’) Forward Primer	(5’-3’) Reverse Primer
NFKBIE (Mus musculus)	[FAM]TATCACCTCTGACCTGGGCTC	TCTGAATGTCCAGGACTTCCTG
NFKBIE (Homo sapiens)	[FAM]TGGAGCCGGATCCTGCTCAG	TAGGGAGCGCAGAGAGCGCA
CXCR4 (Homo sapiens)	[FAM]TCTTAACTGGCATTGTGGGCAAT	TCAACTGCCCAGAAGGGAAGC
CD19 (Homo sapiens)	[FAM]TCCTCGCCTCCTCTTCTT	TTTCCAGCCTCAATCCCTTC

SUPPLEMENTARY MATERIALS AND METHODS

In vivo adoptive transfer experiments

All animal procedures were performed in accordance with Italian legislative decree 26/2014 and European directive 2010/63/EU and were conducted under a protocol approved by the Italian Ministry of Health (no. 218/2022-PR). The CRISPR/Cas9-edited Eμ-TCL1 leukemia cells were transferred into 2-3 months old C57BL/6 or NSG mice via intraperitoneal injection of 3-5x10^7 cells. Leukemia expansion was monitored by WBC counts and CD5/CD19 staining.

For the in vivo ibrutinib treatment experiment, two groups of 2-3 months old female C57BL/6 mice (n=8/group) were treated with vehicle control (1% β-Cyclodextrin) or ibrutinib (25 mg/kg/day) administered in two divided doses by intraperitoneal injection. The number of mice per group was chosen using the software G* Power 3.1.9.2 (Heinrich-Heine-Universität Düsseldorf) based on the estimate that a sample size of 8 animals/per group will provide at least 80% power to detect a more than 30% increase in NFKBIE mutant allele frequency in mice receiving ibrutinib compared to vehicle treatment at a one-sided α level of 0.05. Mice were distributed into the control and ibrutinib-treatment groups by random selection prior to adoptive tumor transfer. No blinding was done.

In vitro and *in vivo* cell proliferation assays

For *in vitro* 5-bromo-2-deoxyuridine (BrdU) staining, 1x10^6 cells were cultured with 10 μM BrdU for the indicated times prior to harvesting. Cells were fixed with 2% paraformaldehyde, permeabilized using 3 M HCl plus 2% Tween 20 solution, neutralized with 0.1 M sodium borate buffer pH 9.0 and stained with anti-BrdU-V450 antibody (BD Horizon, Cat# 563445). For *in vivo* BrdU labeling, 10 mg/mL BrdU (BD Biosciences) in phosphate-buffered saline (PBS) was injected intraperitoneally and 14 hours later leukemia cells from peritoneal cavity and spleen were collected for BrdU incorporation analysis.

Purification of murine CLL cells and DNA isolation

For DNA isolation, murine CLL cells were purified from different anatomical compartments (PC, PB or spleen) using the EasySep Mouse CD19 Positive Selection Kit (Stem Cell Technologies, Cat# 18954).

For BrdU incorporation experiments, the leukemic cells were purified using the EasySep Mouse Pan-B Cell Isolation Kit (Stem Cell Technologies, Cat# 19844).

DNA was extracted using the KAPA Express Extract kit (Sigma Aldrich) following the protocol provided by the manufacturer.

Flow cytometry analysis of murine CLL cells

Single cell suspensions were prepared from spleens by grinding the tissue through 70 μm cell strainers (BD Biosciences) and lysing the red blood cells with ACK-lysing buffer (Thermo Fisher Scientific, Cat# A1049201). Cells from peritoneal cavity were obtained by lavage and were not treated further. Staining of cell surface proteins was performed in PBS with 1% fetal calf serum and 0.02% sodium azide for 30 min at 4 °C. The following antibodies were used: CD5-anti-mouse-PE (BD Biosciences, clone 53-7.3, Cat# 553023), CD19-anti-mouse-APC (Biolegend, clone 1D3/CD19, Cat# 152409), CD19-anti-mouse-BV421 (Biolegend, clone 6D5, Cat# 115537), CD19-anti-mouse-PE (BD Biosciences, clone 1D3, Cat# 557399), CD11b-anti-mouse-APC (Biolegend, clone M1/70, Cat# 101211), F4/80-anti-mouse-FITC (Biolegend, clone BM8, Cat# 123107), CD45.2-anti-mouse-BV421 (BD Horizon, clone 104, Cat# 562895), CD45-anti-mouse-APC (BD Biosciences, clone 30-F11, Cat# 559864), CD45-anti-mouse-APC/Fire 750 (Biolegend, clone 30-F11, Cat# 103153), CD3-anti-mouse-PE (Biolegend, clone 17A2, Cat# 100205), CD3ε-anti-mouse-PerCp/Cyanine 5.5 (Biolegend, clone 145-2C11, Cat# 100327), CD4-anti-mouse-FITC (Biolegend, clone RM4-4, Cat# 116003), CD8a-anti-mouse-APC (Biolegend, clone 53-6.7, Cat# 100711), CD8a-anti-mouse-BV605 (Biolegend, clone 53-6.7, Cat# 100743), CD44-anti-mouse-PE (Biolegend, clone IM7, Cat# 103007), CD62L-anti-mouse-Alexa Fluor 700 (Invitrogen, clone MEL-14, Cat# 56-0621-82), PD-1-anti-mouse-BV421 (Biolegend, clone 29F.1A12, Cat# 135217), PD-L1-anti-mouse-PerCp-eFluor710 (Invitrogen, clone MIH5, Cat# 46-5982-82), Lag3-anti-mouse-BV650 (Biolegend, clone C9B7W, Cat# 125227), TIGIT-anti-mouse-BV421 (Biolegend, clone 1G9, Cat# 142111), Tim3-anti-mouse-BV785 (Biolegend, clone RMT3-23, Cat# 142111), anti-mouse CD16/32 (Biolegend, clone 93, Cat# 101319), LIVE/DEAD Fixable Aqua Dead Cell Stain Kit (Invitrogen, Cat# L34965).

Detection of NFKBIE mutations in patient CLL cells by next generation sequencing (NGS)

All the samples enrolled in this study were sequenced and analyzed with the same pipeline at the Clinical and Experimental Onco-Hematology Unit (Aviano, Italy). Briefly, analysis of NFKBIE mutations was performed by NGS with an amplicon-based strategy covering exons 1 and 2 (ANGSD (RRID:SCR_021865). Specific primers were designed with the Primer3 program and modified according to the Illumina protocol. Amplicon libraries were generated using a modified Illumina protocol starting from 40 ng of DNA (~6,000 diploid genomes), a quantity sufficient to successfully detect mutations at a 1% mutant allele frequency. Multiplex PCR products were generated using Phusion High-Fidelity DNA Polymerase (Thermo Scientific) and subsequently tagged with specific index according to modified procedures for NexteraXT (DNALibrary Preparation kit, Illumina). Purified libraries were pooled and paired-end sequenced in a MiSeq instrument (Illumina). FASTQ files were aligned to the Hg19 reference with Burrows-Wheeler Aligner (BWA)-MEM algorithm, and allele variants were called with Somatic Variant Caller (MiseqReporter). A coverage ≥2,000X was obtained for each sample in 100% of the analyzed sequences.

Flow cytometry analysis of human CLL samples

Flow cytometry analysis in primary peripheral blood CLL samples was done on thawed PBMC, stained with anti-CD19 FITC (BD Biosciences, clone HIB19, Cat# 555412), anti-CD3 BUV395 (BD Biosciences, clone UCHT1, Cat# 564001), anti-CD4 BUV737 (BD Biosciences, clone SK3, Cat# 564305) and anti-CD8 BUV805 (BD Biosciences, clone SK1, Cat# 612889) mAbs, with 7-AAD (BD Biosciences, Cat# 559925) to exclude dead cells, and with anti-TIGIT PE (BD Biosciences, clone TgMab-2, Cat# 568672) and anti-PD-1 BV650 (BD Biosciences, clone EH12.1, Cat# 564104). Cells were analyzed on a FACS Fortessa flow cytometer using FACS DIVA software (BD Biosciences) upon instrument calibration with CS&T beads (Cat# 655051, BD Biosciences). TIGIT and PD-1 data were expressed as percentage of positive cells.

Immunoblotting analysis

For analysis of total protein extracts, cells were washed in ice-cold PBS, pelleted, and lysed in a protease- and phosphatase-inhibiting RIPA lysis buffer (10 mM Tris-HCl, pH 7.4, 5 mM EDTA, 150 mM NaCl, 0.1% SDS, 0.1% sodium deoxycholate) (Sigma-Aldrich). Total cell lysates were kept on ice for 30 minutes, mixed every 10 minutes, and then centrifuged in a benchtop centrifuge (Eppendorf) at 16.000xg for 20 minutes at 4°C. For nuclear translocation assay, nuclear and cytoplasmic protein

extracts were extracted with NE-PER Nuclear and Cytoplasmic Extraction Reagents as per protocol (Thermo Scientific, Cat# 78833). The RC DC Protein Assay (Bio-Rad) was used to quantify the protein concentration within each cell lysate. The protein samples were separated by SDS-PAGE and transferred on Immobilon-P polyvinylidene difluoride membranes (Millipore). Membranes were then blotted at 4°C in the presence of 5% non-fat dry milk with the following antibodies: anti-NFKBIE (Sigma Aldrich, Cat# HPA005941), β-actin (Cell Signaling, Cat# 3700), c-Rel (Cell Signaling, Cat# 12707S), NF-kappaB p65 (Cell Signaling, Cat# D14E12), NF-kappaB2 p100/p52 (Cell Signaling, Cat# 4882S), NF-kappaB1 p105/p50 (Cell Signaling, Cat# 13586S), PARP (Cell Signaling, Cat# 9542S), and GAPDH (Santa Cruz, Cat# sc-365062). Immunodetection was done on an ALLIANCE LD2 chemiluminescence Imaging System (Cleaver Scientific Ltd., Warwickshire, UK), using ECL Plus enhanced-chemiluminescence detection reagents (GE Healthcare, Chicago, IL, USA).

RNA preparation and sequencing

Total RNA was extracted with RNeasy Micro Kit (Qiagen, Cat# 74004) following the manufacturer's recommendations. RNA integrity was examined with the Bioanalyzer 2100 (Agilent). RNA sequencing was performed by Macrogen, Inc. The RNA-seq libraries were prepared according to the standard Illumina protocol with the mRNA-seq Illumina TruSeq. cDNA libraries were checked for quality and quantified using the DNA-1000 kit (Agilent) on a 2100 Bioanalyzer. Each library was sequenced with the NovaSeq 6000 sequencer to obtain 2x100bp reads. Raw data are available at Gene expression database (GEO), accession number GSE231799.

Bioinformatics analysis

Quality of raw sequence files was checked via FastQC (RRID:SCR_014583). Transcript quantification was conducted with SALMON (version v1.8) using Transcriptome index with decoys, starting from GRCm39 genome version (accessed on June 2022). The generated transcript counts were analyzed using DESeq2 package (RRID:SCR_000154). The normalized count matrix (obtained from variance stabilizing transformation (VST) method as implemented in DESeq2 package) was used to explore high-dimensional data property with Principal Component Analysis coupled with a dimensionality reduction algorithm used in the DESeq2 package. Differentially expressed transcripts (DETs) were selected with a p-adjusted cut-off of 0.05 and a log2 Fold Change value greater than 1 (up-regulated

DETs) or lower than -1 (down-regulated DETs). P-value was adjusted for multiple testing using the Benjamini–Hochberg (BH) correction with a false discovery rate (FDR) ≤ 0.05. DETs were then analyzed with a hierarchical clustering method, using correlation distance. Visualization of log2-normalized values and clustering was done using the ComplexHeatmap package (RRID:SCR_017270), while visualization of DETs in volcano plots was done using the EnhancedVolcano package. Functional annotation was performed for all the comparisons and for feature lists of interest. In particular, clusterProfiler package was utilized for Gene Ontology analysis, whereas Reactome and gProfiler2 were used for all other databases.

Statistical analysis

Data are expressed as means ±SD. Differences in leukemia cell numbers, % proliferating cells, % viable cells, and mutant allele frequency were assessed using the t test, paired t test, Mann-Whitney rank sum test, Wilcoxon signed-rank test, or one-way ANOVA, as indicated in the figure legends. Overall survival was computed from date of ibrutinib start to death (events) or last follow-up (censoring). Survival curves and medians were calculated with the method of Kaplan-Meier. The log-rank test was used to compare differences between estimated survival curves. Progression was calculated from the date of ibrutinib treatment initiation to progression (event) or last follow-up (censoring). To account for the competing risk of death, progression was evaluated through cumulative incidence and differences across NFKBIE-mutation groups were tested using Gray's test. The SigmaStat Version 3.1 programme was used for all other statistical analyses (Systat Software). P values are shown in figures. Error bars represent standard deviation.

Data availability

All data from this manuscript are available in the published article and its online supplemental material or in the GEO repository under accession number GSE231799.
